# Evaluation of Agar Dilution Method in Susceptibility Testing of Polymyxins for Enterobacteriaceae and Non-Fermentative Rods: Advantages Compared to Broth Microdilution and Broth Macrodilution

**DOI:** 10.3390/antibiotics11101392

**Published:** 2022-10-11

**Authors:** Xinxin Hu, Lilan Sun, Tongying Nie, Yan Yang, Xiukun Wang, Jing Pang, Xi Lu, Xue Li, Yun Lu, Congran Li, Xinyi Yang, Yao Meng, Guoqing Li, Xuefu You

**Affiliations:** Beijing Key Laboratory of Antimicrobial Agents, Institute of Medicinal Biotechnology, Chinese Academy of Medical Sciences and Peking Union Medical College, Beijing 100050, China

**Keywords:** polymyxins, antimicrobial susceptibility testing, agar dilution, broth microdilution

## Abstract

An accurate and reliable susceptibility testing method for polymyxins is urgently needed not only for the clinical laboratory but also for new polymyxin-like lipopeptide development. Reference broth microdilution (rBMD), which was the recommended method by CLSI-EUCAST in clinics, has been proven not to be ideal, while the agar dilution (AD) method that was widely used in new antibiotics discovery has been neglected. In the present study, the AD method was compared with rBMD and broth macrodilution (BMAD) in susceptibility testing of polymyxin B and colistin against >200 Gram-negative isolates. AD showed strong agreement with BMAD for colistin (except for *Klebsiella aerogenes* and *Pseudomonas aeruginosa*); however, its performance was poor for polymyxin B or compared to rBMD. MICs of AD method were not affected when different types of Petri dishes were used, while glass-bottom microtiter plates could lower the MIC of polymyxins 2–8 times compared to tissue-culture-treated polystyrene plates when using rBMD, which demonstrated that tissue-culture-treated plates were not suitable. It was then validated with non-tissue-culture-treated plates. The culture volume was another influencing factor of accuracy for rBMD, and 200 μL seemed to be the most suitable volume for MIC detection of polymyxins. Additionally, no lack of growth phenomenon (skipped well) was observed for AD when it frequently occurred for both BMAD and rBMD. As for strains carrying *mcr-1* gene, 100% of AD results were in essential agreement (EA) and categorical agreement (CA) with both rBMD and BMAD. Overall, rBMD is convenient and widely accepted for susceptibility testing of polymyxins. Although it may be too early to say that AD is superior compared to rBMD and BMAD, it did show some advantages in repeatability and anti-interference ability.

## 1. Introduction

Polymyxins represent a class of non-ribosomal synthesized, cationic, cyclic-lipopeptide antibiotics that can interact with the lipid A moiety of lipopolysaccharide [1,2]. To date, nine polymyxin superfamily members have been identified: polymyxins A, B, C, D, E, F, K, M, P, S, and T [3,4]. Polymyxin B and colistin (polymyxin E) are the only polymyxins available in clinical practice [5], which were discovered in the 1940s from the soil bacterium *Paenibacillus polymyxa* [6] and were widely used to treat severe infections till the mid-1980s. The rate of polymyxin-associated neurotoxicity and nephrotoxicity was reported as high as 27% and 60% then, which restricted their use. However, neurotoxicity was proved not to be a major concern according to later studies, and nephrotoxicity was reversible in most patients and can be managed through close monitoring [7,8]. Over the last decades, with the growing prevalence of multidrug-resistant bacteria, the paucity or unbearable high cost of new effective antibiotics, polymyxins have re-emerged as the last resort therapeutic option. Although there are international consensus guidelines for use and susceptibility testing of colistin, improvements to susceptibility testing are still required.

Low accuracy and reliability of polymyxin susceptibility testing led to misuse of polymyxins and hindered the development of new polymyxin drugs [9,10]. In 2016, a CLSI-EUCAST working group recommended ISO-standard BMD (20776-1) as the reference method when testing MIC of colistin on *Enterobacteriaceae*, *P. aeruginosa*, and *Acinetobacter spp.* [11]. However, adherence of polymyxin molecules onto the plastic-surface plates used in BMD has raised concerns about the reliability and inter-laboratory comparison of MIC results [12]. As for the AD and BMAD methods, there were relatively few studies to analyze their performance [13,14,15,16,17,18]. Thus, there is still a long way to go to find a practical method for polymyxin susceptibility testing. In the current work, we compared the AD with the rBMD and BMAD methods to determine the feasibility of AD to be used in the susceptibility testing of polymyxins.

## 2. Results

### 2.1. MICs of Polymyxin B and Colistin by Three Susceptibility Methods

220 Gram-negative isolates, including 40 *E. coli*, 38 *Klebsiella pneumoniae*, 37 *Enterobacter cloacae*, 30 *K. aerogenes*, 38 *Acinetobacter baumannii*, and 37 *P. aeruginosa,* were tested. The MICs of quality control strains were within the acceptable range for all three methods. The MIC_50_ and MIC_90_ are shown in Table 1; MIC distributions for polymyxin B and colistin are presented in Supplementary Materials Tables S1–S3. Generally, AD resulted in moderate MICs which were lower than rBMD but higher than BMAD for both polymyxin B and colistin. The MIC_50_ ranges were 0.25–4.0 μg/mL for AD, 0.5–8.0 μg/mL for rBMD, and 0.125–4.0 μg/mL for BMAD.

Of the 220 isolates, only a single strain (0.5%) was resistant to polymyxin B and all isolates were sensitive to colistin when tested by AD; the corresponding MICs were in the ranges of 0.5–4.0 μg/mL and 0.25–2.0 μg/mL, respectively (Supplementary Material Table S1). Of these, 27 (12%) were resistant to polymyxin B and 20 (9%) to colistin for rBMD method; the corresponding MICs ranged around 0.5–8.0 μg/mL and 0.5–4.0 μg/mL (Supplementary Material Table S2). As for BMAD, 3 (1%) strains were resistant to polymyxin B and 8 (4%) to colistin, with corresponding MICs ranging from 0.25–4.0 μg/mL and 0.125–4.0 μg/mL (Supplementary Material Table S3).

As for 7 *mcr-1*-positive strains (not included in the 220 isolates) which were used to test the impact of resistance genes carried by plasmids, the MICs (Table 2) showed that AD, rBMD, and BMAD correlated very well with each other (100% EA and 100% CA, no VME or ME). All strains were resistant to both polymyxins B and colistin using AD, rBMD, and BMAD; MICs ranged from 4–16 μg/mL, 4–8 μg/mL, and 4–8 μg/mL for polymyxin B, respectively, and 8–32 μg/mL, 8–16 μg/mL, and 8–32 μg/mL for colistin, respectively.

### 2.2. Comparison of AD with Other Susceptibility Testing Methods

**Figure 1:**
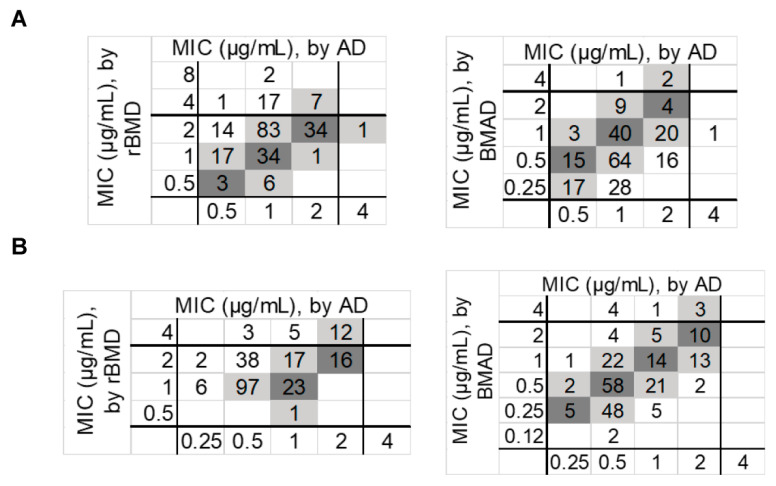
Scatterplot of MIC values for polymyxin B (**A**) and colistin (**B**) measured by AD versus rBMD, and AD versus BMAD using all study isolates (*n* = 220). The coordinate axis shows the MIC range of the test method. Dark gray, absolute agreement; light gray, essential agreement. BMAD: broth macrodilution; rBMD: reference broth microdilution; AD: agar dilution.

**Comparison to rBMD as the test method.** The comparative result between AD and rBMD is shown in Table 3 and Figure 1. The correlation of AD and rBMD was especially poor, which revealed VMEs of 12.2% and 9.1% for polymyxin B and colistin, respectively. Acceptable MEs of polymyxin B (0.4%) and colistin (0%) were observed. CA for polymyxin B (87.3%) was a little bit lower than that of colistin (90.9%), whereas EAs of 85% and 75.1% were observed for polymyxin B and colistin, respectively. It was worthy of note that VMEs for *P. aeruginosa* were 29.7% and 35.1%, respectively, for polymyxin B and colistin.

**Comparison to BMAD as the test method.** Overall, AD showed a high level of agreement with BMAD for colistin when testing *E. coli* and *A. baumannii*, and the results of *K. pneumonia* and *E. cloacae* were barely acceptable. However, the VMEs of *K. aerogenes* (6.7%) and *P. aeruginosa* (10.8%) were extremely high. Although the overall VME, ME and CA were within the acceptance criteria for polymyxin B, the EA was much too low (79.1%).

### 2.3. Differences of MICs between Polystyrene and Glass-Bottom Microtiter Plates

**Figure 2:**
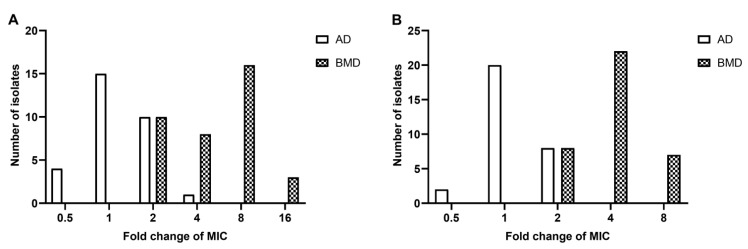
Comparison of MIC values for polymyxin B (**A**) and colistin (**B**) using polystyrene and glass plates. Fold change of MIC means the ratio of the MIC values obtained from polystyrene to those obtained from glass plates (AD: non-tissue-culture-treated plates vs. glass plates; rBMD: tissue-culture-treated microtiter plates vs. glass-bottom microtiter plates).

It was noted that glass-bottom microtiter plates would bring 2–8 times lower MICs of polymyxin B and colistin than tissue culture-treated polystyrene plates for all 6 isolates tested (Table 4). The results indicate that polymyxins were adsorbed onto surface of the plastic plates, which led to a higher MIC; whereas the degrees of polymyxin loss were much lower in glass-bottom plates. When using non-tissue culture-treated polystyrene plates, the MICs correspond well with glass-bottom ones (Table 4). When comparing MICs of tissue culture-treated polystyrene and glass-bottom plates with 37 clinical isolates, it validated what we saw in standard strains, i.e., that tissue culture-treated polystyrene plates were unreliable for rBMD (Figure 2).

To further investigate whether the adsorption of polymyxins was affected by the culture volume, two types of 96-well plates (glass-bottom and tissue-culture-treated polystyrene plates) and three different volumes were used to determine MICs of polymyxin B and colistin (Table 5). Compared with 100 μL culture volume, the MICs of polymyxins were 2–4 times lower when using 200 μL or 300 μL broth per well, while the MICs of levofloxacin (negative control) were identical in all three volumetric systems. All of these results revealed that as the volume of the test system increased, the loss of polymyxins due to adsorption decreased, and the measured MICs were more accurate. Hence, when rBMD is applied for polymyxins MIC determination, reliability can be improved by using a 200 uL culture volume.

### 2.4. Differences of MICs between Polystyrene and Glass Petri Dishes

Testing was conducted with 6 ATCC isolates to compare the MICs between glass, tissue-culture-treated and non-tissue-culture-treated polystyrene dishes. The results showed that MICs differed within two-fold, which were in acceptable ranges [19,20] (Table 4). MICs of non-tissue-culture-treated polystyrene dishes and glass dishes were then compared with 30 clinical isolates. It showed that most isolates exhibited the same MICs on two types of dishes, and MICs of the remaining strains differed by an acceptable two-fold only (Figure 2). Overall, AD showed great consistency on different materials or types of dishes.

### 2.5. The Ratio of Lack of Growth

Lack of growth phenomenon is equivalent to skip well for rBMD and BMAD, which means bacteria exhibit no growth at a lower antibiotic concentration, whereas growth is observed with higher concentrations [9]. In our studies, no lack of growth occurred for AD, while skip wells in 4 of 220 strains for polymyxin B and 9 of 220 strains for colistin when testing with rBMD and in 17 of 220 strains for polymyxin B and colistin when testing with BMAD were observed.

## 3. Discussion

Although several potent antibiotics (such as cefiderocol and meropenem–vaborbactam) were recommended to deal with severe Gram-negative infections, polymyxins remain to be the only option for infections caused by carbapenem-resistant isolates in many countries where these costly new antibiotics are unavailable [21]. Colistin also has an important role as the salvage therapy for cystitis and HAP/VAP, which is endorsed by Infectious Diseases Society of America (IDSA), European Society of Clinical Microbiology and Infectious Diseases (ESCMID), and Chinese Research Hospital Association of Critical Care Medicine [22,23,24]. Therefore, optimization and standardization of in vitro polymyxin susceptibility testing, as well as the definition of accurate breakpoints, are critical issues for both patient care and epidemiological surveillance purposes, particularly in view of the increased clinical use of polymyxins.

CLSI and EUCAST had a consensus that rBMD should be used as a reference method, but they had no agreement on some other issues. For CLSI, rBMD, CBDE, and CAT, MIC methods are all acceptable now for colistin and polymyxin B when testing *Enterobacterals* and *P. aereginosa*, but rBMD is the only approved method for *Acinetobacter spp.* [25]. In contrast to CLSI, ECUAST only approved rBMD method for colistin susceptibility testing [26]. CLSI deleted the breakpoint of susceptible category (S) after reviewing the preclinical PK/PD, clinical PK/TD, and MIC distribution data, and classified the strains with MIC ≤ 2 μg/mL into intermediate category in 2020, while EUCAST maintains ≤2 μg/mL as the breakpoint of the susceptible category.

Numerous studies have shown that commercial disk diffusion, gradient strips, and automated detection systems were unreliable for assessing colistin susceptibility, exhibiting unacceptable high rates of very major errors (VME) and low levels of reproducibility [1]. rBMD is the only co-validated susceptibility testing method for polymyxins by CLSI and EUCAST, although it is not an ideal one. Mariana et al. reported that when retesting 200 *K. pneumonia* with rBMD, 40% of the MICs showed differences greater than the ±1 dilution accepted variability, indicating the low reproducibility of rBMD [27]. Romney et al. found CAMHB from Oxoid would yield unacceptable low MICs for QC strain *P. aeruginosa* ATCC 27853 [28]. We used Corning Costar^TM^ 3799 plates for rBMD, and MICs of the QC strains were all within the QC range. It is the most commonly used plate for rBMD, but Mahablleshwar et al. discovered that this type of plate, which are treated with corona discharge (so-called tissue-culture-treated), yielded a much higher MIC comparing to non-coated ones [29]. Then we compared the MIC results of Costar^TM^ 3799 plates with glass-bottom plates and non-tissue culture-treated plates and found that the MICs of the latter two plates were 2–4 times lower, which meant a low adsorption rate. It demonstrated that tissue culture treatment played an important role in binding ability of polymyxins to plate surface, and the current QC range is not appropriate [30]. On the other hand, neither CLSI nor EUCAST specified the types or brands of microtiter plates that should be chosen for rBMD, which may cause many uncertainties and problems [25,26]. In some published literature, the authors used customized rBMD panels that could ensure accurate results [31,32], while the tissue-culture-treated plates may be misused to get higher MICs with rBMD [33]. The adsorption of polymyxins to plastic plates has been widely discussed; Matti et al. characterized the extent of colistin loss in plates of different types and brands [12]. They found both polystyrene and polypropylene microtiter plates could adsorb colistin in different intensities; in addition, two brands of plates yielded a significant difference in the measured concentrations. Low-protein-binding polypropylene plates or glass-coated plates (similar to glass-bottom plates) were recommended for measuring MIC of colistin, but the cost of these plates is much too high [1,12].

Furthermore, we measured the MICs with polystyrene and glass-bottom plates using different volumes of broth and found out the MICs would decrease with larger culture volume regardless of types of the plates. It could be explained by the adsorption characteristics of polymyxins because the surface area to volume ratio of 100 μL system is bigger than the 200 μL one. In addition, the surface-area-to-volume ratio of AD on 9 cm Petri dishes is close to 200μL system, which could explain the comparability of the results of these two methods. CLSI and EUCAST recommended 100 μL as the standard volume of rBMD, while in our opinion, 200 μL seems to be better considering the capacity of the microtiter plates.

AD is thought to be the solid equivalent of broth dilution, either in the rBMD or BMAD format [34], which relies on various concentrations of 2-fold dilution of antibiotics in agar plates. AD is rarely used because it is time- and labor-consuming compared to Etest and disk diffusion in clinical use, although it could test as many as 37, 60, 70, or even more isolates on one plate simultaneously with a semi-auto multipoint inoculator. The CAT method, which is approved by CLSI in 2020, is actually a modified AD method [35]. Operators need to streak 10 μL suspension onto an agar plate with a pipette or loop instead of multipoint inoculators which are uncommon in clinical use. The AD method, consistent with CAT, has no diffusion problem as Etest and disk diffusion and may theoretically avoid the adsorption of polymyxins to the plate surface [1], which was proved by our experiments. Further, Fereshteh et al. reported that the MICs showed no difference when testing ATCC strains with 1-week-old colistin agar plate or freshly made ones [36]. We also noted that no lack of growth phenomenon was observed for AD, whereas it happened at 1.8% or 4.1% for rBMD when testing polymyxin B or colistin, respectively, and 7.7% for BMAD when testing polymyxin B and colistin. It is related to heteroresistance [37], and small inoculation volume (1–2 μL for AD, 10 μL for rBMD and 100 μL for BMAD) may be the reason for low incidence [1]. We could not say it is an advantage of AD, but it did facilitate the reading and results analysis.

Binding of polymyxins to labware is concentration-dependent and saturable [38]. It has been proved that the proportion of free colistin would decrease with lower drug concentration. The same conclusion has been revealed from our result. We tested all the isolates with AD, rBMD, and BMAD and found out that only at high concentrations of polymyxins (≥4 μg/mL) when binding was saturated, the MICs of these three methods could agree well with each other.

CLSI and EUCAST considered colistin and polymyxin B as equivalent, and the MIC of one agent could predict that of the other one, but our result showed MICs of colistin was lower than polymyxin B, indicating a stronger activity of colistin.

## 4. Materials and Methods

### 4.1. Bacterial Strains

Briefly, 212 clinical isolates collected from Peking Union Medical College Hospital and The First Affiliated Hospital of Hebei North University and 8 strains from the American Type Culture Collection (ATCC) were used to evaluate the antibacterial susceptibility of colistin and polymyxin B with three testing methods: AD, rBMD, and BMAD. The characteristics of the isolates used were summarized in Supplementary Material Table S4. To evaluate the impact of resistance genes carried by plasmids, 7 *mcr-1*-positive strains not included in the 220 isolates (1 standard strain NCTC 13846, 5 *E. coli* clinical isolates, and 1 *K. pneumonia* clinical isolate) were tested. All strains were stored at the CAMS Collection Center of Pathogen Microorganisms (CAMS-CCPM) in Beijing, China.

### 4.2. Antibacterial Agents and Susceptibility Testing

Polymyxin B sulfate and colistin sulfate were purchased from the National Institutes for Food and Drug Control (Beijing, China). The powdered drugs were dissolved with sterilized double-distilled water into a concentration of 10 mg/mL and stored at −80 °C until use. rBMD followed the CLSI guidelines, AD and BMAD were performed according to the existing procedures [39]. Tubes and plates were incubated at 35 ± 2 °C for 16–18 h, followed by a visual assessment of turbidity. *E. coli* ATCC 25922 and *P. aeruginosa* ATCC 27853 were used as quality control strains in the susceptibility tests.

**Agar dilution.** Difco^TM^ Mueller–Hinton agar (BD, Franklin Lakes, NJ, USA) plates containing 0.125–256 μg/mL polymyxins were prepared in Petri dishes. A 1 μL inoculum of 1:10 dilution of a 0.5 McFarland suspension was inoculated onto agar plates using a Denley^®^ (Denley Instruments Ltd, Sussex, UK) multipoint inoculator (37 spots per plate). The final bacterial inoculum amounted to 1 × 10^4^ CFU/spot. Non-tissue culture-treated polystyrene dishes are most accessible; therefore, experiments were carried out on this type of dish. To investigate the variance of binding ability of different materials, glass and tissue-culture-treated polystyrene dishes were used, and results were compared.

**Broth dilution.** To minimize the loss of polymyxins in dilution procedure, incremental dilution was carried out. Briefly, polymyxin B and colistin stock solutions were diluted into working solutions (0.125 μg/mL to 16 μg/mL) using BBL^TM^ cation-adjusted Mueller–Hinton II broth (BD, Franklin Lakes, NJ, USA). Antibiotic concentrations were the same for BMAD and rBMD, and 1 mL or 100 μL of each dilution was dispensed to glass tubes or microtiter plates. Bacterial suspensions were adjusted to 0.5 McFarland and the final inoculum amounted to 5 × 10^5^ CFU/mL for both methods. Tissue-culture-treated microtiter plates were from Corning (Costar^TM^ 3799; Corning, ME, USA), which are the most commonly used plates for rBMD in China and some other countries. To further explore the binding ability of polymyxin to the surface of microtiter plates, glass-bottom plates (Cellvis, Mountain View, CA, USA) and non-tissue-culture-treated plates (Nest, China) were used for comparison.

### 4.3. Interpretation of Antimicrobial Susceptibility Testing Methods

EUCAST-recommended MIC breakpoints for colistin were used to assess differences between the tested methods. For *Enterobacteriaceae*, *P. aeruginosa*, and *Acinetobacter spp.*, EUCAST recommends a breakpoint ≤2 μg/mL for susceptible and ≥4 μg/mL for resistant (EUCAST, 2021). CLSI mandates the quality control range of polymyxin B and colistin for *E. coli* ATCC 25922 to be 0.25–2.0 μg/mL, while for *P. aeruginosa* ATCC 27853 those values are 0.5–2.0 μg/mL and 0.5–4.0 μg/mL.

Testing would be repeated for isolates when >1 skipped well occurred with broth dilution method, and if it happened again, the isolates would be excluded. It was the same for AD when isolates did not grow on low concentration plates but grew on plates with higher concentrations of polymyxins.

### 4.4. Data Analysis

MIC_50_ and MIC_90_ values were calculated for all susceptibility testing methods. Essential agreement (EA) and categorical agreement (CA) were evaluated; very major errors (VME) and major errors (ME) were analyzed. EA was defined as MICs of the two methods were within a single doubling dilution; CA was the proportion of isolates classified in the same susceptibility category by the methods evaluated; VME was defined as that the result of the test method was susceptible while the result of the reference method was resistant; ME was defined as that the result of the test method was resistant while the result of the reference method was susceptible.

## 5. Conclusions

The rBMD method which CLSI-EUCAST recommended for susceptibility testing of polymyxins is convenient and widely accepted, although it has quite a lot of limitations. It is still challenging for both clinical and research labs to carry out the experiment without an ideal method. According to the results of this study, we think the reference method should be improved further, such as clearly specifying the brand of CAMHB and plate, using 200 μL culture volumes, and reevaluating the interchangeability of polymyxin B and colistin. BMAD developed by Fleming a century ago is a traditional method. It can be seen as the enlarged BMD except that it is very laborious. AD has some advantages in repeatability and anti-interference ability compared to rBMD and BMAD, but it is time- and labor-consuming and is rarely used by clinical laboratories. All in all, the feasibility to use AD as a reference method is still an open question.

## Figures and Tables

**Table 1:** MIC_50_ and MIC_90_ of polymyxins.

Species	PMB						CST					
	AD		rBMD*		BMAD		AD		rBMD*		BMAD	
μg/mL	MIC_50_	MIC_90_	MIC_50_	MIC_90_	MIC_50_	MIC_90_	MIC_50_	MIC_90_	MIC_50_	MIC_90_	MIC_50_	MIC_90_
*E. coli*	0.5	1	1	2	0.5	1	0.5	0.5	1	2	0.25	0.5
*K. pneumoniae*	1	2	2	2	1	2	0.5	1	1	2	0.5	1
*E. cloacae*	1	1	2	4	0.5	1	1	1	1	2	0.5	1
*K. aerogenes*	1	1	2	2	0.5	0.5	0.5	0.5	1	2	0.25	0.5
*A. baumannii*	1	1	2	2	0.5	1	0.5	0.5	1	2	0.5	1
*P. aeruginosa*	2	2	2	4	1	1	2	2	2	4	1	2

PMB: polymyxin B; CST: colistin; AD: agar dilution; rBMD: reference broth microdilution; BMAD: broth macrodilution. *: using tissue-culture-treated microtiter plates.

**Table 2:** MIC values (μg/mL) of polymyxin B and colistin for *mcr-1* positive isolates.

No.	Strains	PMB			CST		
		AD	rBMD*	BMAD	AD	rBMD*	BMAD
1	*E. coli* NCTC13846	4	4	4	8	8	8
2	*E. coli* CCPM(A)-P-070885	8	4	4	16	16	8
3	*E. coli* CCPM(A)-P-071343	8	4	4	16	16	8
4	*E. coli* CCPM(A)-P-071366	8	4	4	16	8	8
5	*E. coli* CCPM(A)-P-071368	8	8	4	8	16	8
6	*E. coli* CCPM(A)-P-0717R14	8	4	4	8	8	8
7	*K. pneumoniae* CCPM(A)-P-080920	16	8	8	32	16	32

No.: number; PMB: polymyxin B; CST: colistin; AD: agar dilution; rBMD: reference broth microdilution; BMAD: broth macrodilution. *: using tissue-culture-treated microtiter plates.

**Table 3:** AD Compared with rBMD and BMAD for polymyxin B and colistin.

Species	Tested Agents	rBMD*				BMAD			
%		VME	ME	CA	EA	VME	ME	CA	EA
*E. coli*	PMB	2.5	0	97.5	80	0	0	100	87.5
*K. pneumoniae*		10.5	0	89.5	94.7	5.3	0	94.7	89.5
*E. cloacae*		10.8	0	89.2	86.5	0	0	100	62.2
*K. aerogenes*		3.3	0	96.7	90	0	0	100	73.3
*A. baumannii*		15.8	0	84.2	76.3	2.6	0	97.4	86.8
*P. aeruginosa*		29.7	2.7	67.6	81.1	0	2.7	97.3	73
**Total (%)**		**12.2**	**0.4**	**87.3**	**85**	**1.4**	**0.4**	**98.2**	**79.1**
*E. coli*	CST	2.5	0	97.5	67.5	0	0	100	92.5
*K. pneumoniae*		7.9	0	92.1	68.4	2.6	0	97.4	86.8
*E. cloacae*		0	0	100	78.4	2.7	0	97.3	89.2
*K. aerogenes*		0	0	100	73.3	6.7	0	93.3	90
*A. baumannii*		7.9	0	92.1	71.1	0	0	100	100
*P. aeruginosa*		35.1	0	64.9	94.6	10.8	0	89.2	89.2
**Total (%)**		**9.1**	**0**	**90.9**	**75.1**	**3.6**	**0**	**96.4**	**91.8**

VME: very major errors; ME: major errors; CA: categorical agreement; EA: essential agreement; PMB: polymyxin B; CST: colistin; BMAD: broth macrodilution; AD: agar dilution; rBMD: reference broth microdilution. *: using tissue-culture-treated microtiter plates.

**Table 4:** Differences of MICs of polymyxin B and colistin on different plate materials.

Tested Agents	Strains	rBMD			AD		
		TC-PS	GB	nTC-PS	TC Plates	GA	nTC Plates
PMB	*E. coli* ATCC 25922	2	0.5	1	2	1	1
	*E. coli* ATCC 2469	2	0.5	0.5	1	0.5	1
	*K. pneumoniae* ATCC 700603	4	0.5	0.5	2	1	1
	*K. pneumoniae* ATCC 2146	4	0.5	0.5	2	1	1
	*A. baumannii* ATCC 19606	2	0.5	0.5	1	0.5	1
	*P. aeruginosa* ATCC 27853	2	0.5	1	2	1	2
CST	*E. coli* ATCC 25922	2	1	1	1	0.5	1
	*E. coli* ATCC 2469	2	0.5	0.5	1	0.5	0.5
	*K. pneumoniae* ATCC 700603	4	0.5	0.5	1	0.5	1
	*K. pneumoniae* ATCC 2146	2	0.5	0.5	1	0.5	1
	*A. baumannii* ATCC 19606	2	1	1	1	0.5	0.5
	*P. aeruginosa* ATCC 27853	2	1	1	2	1	1

PMB: polymyxin B; CST: colistin; TC-PS: tissue-culture-treated polystyrene microtiter plates; GB: glass-bottom microtiter plates; nTC-PS: non-tissue-culture-treated polystyrene microtiter plates; TC plates: tissue-culture-treated polystyrene plates; GA: glass plates; nTC plates: non-tissue culture-treated polystyrene plates; rBMD: reference broth microdilution; AD: agar dilution.

**Table 5:** MIC values (μg/mL) measured with three volumes and two plate materials by rBMD.

Tested Agents	Strains	100 μL		200 μL		300 μL	
		TC-PS	GB	TC-PS	GB	TC-PS	GB
PMB	*E. coli* ATCC 25922	2	0.5	1	0.25	1	0.25
	*E. coli* ATCC 2469	2	0.5	0.5	0.25	0.5	0.12
	*K. pneumoniae* ATCC 700603	4	0.5	1	0.25	1	0.25
	*K. pneumoniae* ATCC 2146	4	0.5	1	0.12	1	0.12
	*A. baumannii* ATCC 19606	2	0.5	1	0.12	1	0.12
	*P. aeruginosa* ATCC 27853	2	0.5	1	0.5	1	0.5
CST	*E. coli* ATCC 25922	2	1	1	0.25	1	0.25
	*E. coli* ATCC 2469	2	0.5	1	0.25	0.5	0.25
	*K. pneumoniae* ATCC 700603	4	0.5	1	0.25	1	0.25
	*K. pneumoniae* ATCC 2146	2	0.5	1	0.12	0.5	0.25
	*A. baumannii* ATCC 19606	2	1	1	0.5	1	0.5
	*P. aeruginosa* ATCC 27853	2	1	1	0.5	1	0.5

PMB: polymyxin B; CST: colistin; TC-PS: tissue-culture-treated polystyrene microtiter plates; GB: glass-bottom microtiter plates; rBMD: reference broth microdilution. Levofloxacin was used as a control and MIC values of levofloxacin were consistent with three volumes and two plate materials.

## Data Availability

Not applicable.

## Supplementary Materials

The following supporting information can be downloaded at: https://www.mdpi.com/article/10.3390/antibiotics11101392/s1, Table S1: MIC distributions of isolates tested by AD method; Table S2: MIC distributions of isolates tested by rBMD method; Table S3: MIC distributions of isolates tested by BMAD method; Table S4: Characteristics of the 220 isolates used in the study.
