# Supplementary material for: Evaluation of Agar Dilution Method in Susceptibility Testing of Polymyxins for Enterobacteriaceae and Non-Fermentative Rods: Advantages Compared to Broth Microdilution and Broth Macrodilution
Source: Antibiotics (Basel). 2022 Oct 11;11(10):1392. doi: 10.3390/antibiotics11101392 (PMC9598209; doi:10.3390/antibiotics11101392)
Supplement: Supplementary file 1 [file antibiotics-11-01392-s001.zip › antibiotics-1926308-supplementary.pdf]

Table S1. MIC distributions of isolates tested by AD method

Species	No.	Tested agents	R	S	MIC range (μg/mL)	MIC (μg/mL)				
						0.25	0.5	1	2	4
E. coli	40	PMB	0	40	0.5-1		19	21		
K. pneumoniae	38		0	38	1-2			31	7	
E. cloacae	37		0	37	0.5-2		5	27	5	
K. aerogenes	30		0	30	0.5-1		4	26		
A. baumannii	38		0	38	0.5-2		7	27	4	
P. aeruginosa	37		1	36	1-4			10	26	1
Total	220		1	219			35	142	42	1
E. coli	40	CST	0	40	0.25-1	5	34	1		
K. pneumoniae	38		0	38	0.5-1		29	9		
E. cloacae	37		0	37	0.25-1	1	32	4		
K. aerogenes	30		0	30	0.25-0.5	1	29			
A. baumannii	38		0	38	0.25-2	1	13	23	1	
P. aeruginosa	37		0	37	0.5-2		1	9	27	
Total	220		0	220		8	138	46	28	

No.: number; R: resistant; S: susceptible; PMB: polymyxin B; CST: colistin; AD: agar dilution

Table S2. MIC distributions of isolates tested by rBMD* method

Species	No.	Tested agents	R	S	MIC range (μg/mL)	MIC (μg/mL)				
						0.5	1	2	4	8
E. coli	40		1	39	0.5-4	1	17	21	1	
K. pneumoniae	38	PMB	4	34	0.5-4	1	12	21	4	
E. cloacae	37		4	33	0.5-4	7	7	19	4	
K. aerogenes	30		1	29	1-4		8	21	1	
A. baumannii	38		6	32	1-8		6	26	4	
P. aeruginosa	37		11	26	1-4		2	24	11	
Total	220		27	193		9	52	132	25	2
E. coli	40		1	39	1-4		31	8	1	
K. pneumoniae	38		3	35	1-4		24	11	3	
E. cloacae	37		0	37	1-2		26	11		
K. aerogenes	30	CST	0	30	1-2		23	7		
A. baumannii	38		3	35	0.5-4	1	19	15	3	
P. aeruginosa	37		13	24	1-4		3	21	13	
Total	220		20	200		1	126	73	20	

No.: number; R: resistant; S: susceptible; PMB: polymyxin B; CST: colistin; rBMD: reference broth microdilution

*: using tissue culture-treated microtiter plates

Table S3. MIC distributions of isolates tested by BMAD method

Species	No.	Tested agents	R	S	MIC range (μg/mL)	MIC (μg/mL)					
						0.125	0.25	0.5	1	2	4
E. coli	40	PMB	0	40	0.25-2		15	17	7	1	
K. pneumoniae	38		2	36	0.25-4		2	15	13	6	2
E. cloacae	37		0	37	0.25-2		10	22	4	1	
K. aerogenes	30		0	30	0.25-2		12	13	4	1	
A. baumannii	38		1	37	0.25-4		5	17	14	1	1
P. aeruginosa	37		0	37	0.25-2		1	11	22	3	
Total	220		3	217			45	95	64	13	3
E. coli	40	CST	0	40	0.125-1	2	19	14	5		
K. pneumoniae	38		1	37	0.25-4		9	14	10	4	1
E. cloacae	37		1	36	0.25-4		19	13	3	1	1
K. aerogenes	30		2	28	0.25-4		9	14	5		2
A. baumannii	38		0	38	0.5-2			23	13	2	
P. aeruginosa	37		4	33	0.25-4		2	5	14	12	4
Total	220		8	212		2	58	83	50	19	8

No.: number; R: resistant; S: susceptible; PMB: polymyxin B; CST: colistin; BMAD: broth macrodilution

Table S4. Characteristics of the 220 isolates used in the study

Species	Phenotype	No.
E. coli	CRE	10
	ESBLs(+)	15
	ESBLs(-)	15
	Total	40
K. pneumoniae	CRE	12
	ESBLs(+)	11
	ESBLs(-)	15
	Total	38
E. cloacae	CRE	12
	Non-CRE	25
	Total	37
K. aerogenes	CRE	11
	Non-CRE	19
	Total	30
A. baumannii	CRAB	10
	Non-CRAB	28
	Total	38
P. aeruginosa	CRPA	6
	Non-CRPA	31
	Total	37

No.: number
